# Attraction and Electrophysiological Response to Identified Rectal Gland Volatiles in *Bactrocera frauenfeldi* (Schiner)

**DOI:** 10.3390/molecules25061275

**Published:** 2020-03-11

**Authors:** Saeedeh Noushini, Jeanneth Perez, Soo Jean Park, Danielle Holgate, Vivian Mendez Alvarez, Ian Jamie, Joanne Jamie, Phillip Taylor

**Affiliations:** 1 Department of Molecular Sciences, Macquarie University, Sydney, NSW 2109, Australia; danielle.holgate@hdr.mq.edu.au (D.H.); ian.jamie@mq.edu.au (I.J.); joanne.jamie@mq.edu.au (J.J.); 2 Australian Research Council Industrial Transformation Training Centre for Fruit Fly Biosecurity Innovation, Macquarie University, Sydney, NSW 2109, Australia; jeanneth.perez@mq.edu.au (J.P.); soojean.park@mq.edu.au (S.J.P.); vivian.mendez@mq.edu.au (V.M.A.); Phil.Taylor@mq.edu.au (P.T.); 3 Applied BioSciences, Macquarie University, Sydney, NSW 2109, Australia

**Keywords:** *B. frauenfeldi*, mango fruit fly, insect volatiles, GC-EAD, olfaction

## Abstract

*Bactrocera frauenfeldi* (Schiner) (Diptera: Tephritidae) is a polyphagous fruit fly pest species that is endemic to Papua New Guinea and has become established in several Pacific Islands and Australia. Despite its economic importance for many crops and the key role of chemical-mediated sexual communication in the reproductive biology of tephritid fruit flies, as well as the potential application of pheromones as attractants, there have been no studies investigating the identity or activity of rectal gland secretions or emission profiles of this species. The present study (1) identifies the chemical profile of volatile compounds produced in rectal glands and released by *B. frauenfeldi*, (2) investigates which of the volatile compounds elicit an electroantennographic or electropalpographic response, and (3) investigates the potential function of glandular emissions as mate-attracting sex pheromones. Rectal gland extracts and headspace collections from sexually mature males and females of *B. frauenfeldi* were analysed by gas chromatography-mass spectrometry. Male rectal glands contained (*E*,*E*)-2-ethyl-8-methyl-1,7-dioxaspiro [5.5]undecane as a major component and (*E*,*E*)-2,8-dimethyl-1,7-dioxaspiro[5.5]undecane as a moderate component. Minor components included palmitoleic acid, palmitic acid, and ethyl oleate. In contrast, female rectal glands contained (*E*,*E*)-2,8-dimethyl-1,7-dioxaspiro[5.5]undecane and ethyl laurate as major components, ethyl myristate and ethyl palmitoleate as moderate components, and 18 minor compounds including amides, esters, and spiroacetals. Although fewer compounds were detected from the headspace collections of both males and females than from the gland extractions, most of the abundant chemicals in the rectal gland extracts were also detected in the headspace collections. Gas chromatography coupled electroantennographic detection found responses to (*E*,*E*)-2,8-dimethyl-1,7-dioxaspiro[5.5]undecane from the antennae of both male and female *B. frauenfeldi*. Responses to (*E*,*E*)-2-ethyl-8-methyl-1,7-dioxaspiro[5.5]undecane were elicited from the antennae of females but not males. The two spiroacetals also elicited electropalpographic responses from both male and female *B. frauenfeldi*. Ethyl caprate and methyl laurate, found in female rectal glands, elicited responses in female antennae and palps, respectively. Y-maze bioassays showed that females were attracted to the volatiles from male rectal glands but males were not. Neither males nor females were attracted to the volatiles from female rectal glands. Our findings suggest (*E*,*E*)-2,8-dimethyl-1,7-dioxaspiro[5.5]undecane and (*E*,*E*)-2-ethyl-8-methyl-1,7-dioxaspiro[5.5]undecane as components of a sex-attracting pheromone in *B. frauenfeldi*.

## 1. Introduction

Many tephritid fruit flies, belonging to the genus *Bactrocera*, are significant horticultural pests, causing direct damage to fruit and impeding trade [1,2,3,4]. The mango fruit fly, *Bactrocera frauenfeldi* (Schiner) (Diptera: Tephritidae), is an economically important tephritid pest, with hosts including guava (*Psidium guajava*), mango (*Mangifera indica*), beach almond (*Terminalia catappa*), and Alexandrian laurel (*Calophyllum inophyllum*) [5,6,7]. Endemic to Papua New Guinea [8], *B. frauenfeldi* has become established on several Pacific Islands, including the Solomon Islands, the Federated States of Micronesia, the Republic of Kiribati, Marshall Islands, Palau, Nauru, West Papua in Indonesia [9,10,11], and in Australia [6,12,13,14,15]. Surveillance, monitoring, and control of tephritid fruit flies involves the use of lures [16]. Food-based lures have been used as attractants in traps for detecting and monitoring tephritid flies [17]. However they are not as powerful as male lures, such as cuelure, zingerone, and methyl eugenol [18]. *Bactrocera frauenfeldi* adult males are highly attracted to cuelure and raspberry ketone, and are weakly attracted to zingerone [15,18]. Raspberry ketone and zingerone are naturally occurring compounds found in many plants [19,20]. Raspberry ketone is also known as a fungal metabolite [21]. While cuelure has not been found in nature, it hydrolyses to raspberry ketone [16]. Ingested raspberry ketone, zingerone, and cuelure accumulate in the rectal gland of some male s of some fruit fly species in their original form, while zingerone and cuelure are also to some extent transformed [22,23,24]. However, similar to other fruit flies, there is a lack of a specific attractant for females of *B. frauenfeldi*.

Volatile compounds produced in the rectal glands of tephritid fruit flies and emitted during calling and courtship have been described as the key elements for long and short-range attraction of the opposite sex [25,26,27,28]. The volatile emissions also play an important role in attracting the same sex to mating aggregations [29,30]. Rectal gland secretions of some fruit fly species have been studied for potential applications as attractants, including *B. tryoni* (Froggatt) [31], *Zeugodacus cucumis* (French) [32,33,34,35], *B. dorsalis* (Hendel) [36], *Z. cucurbitae* (Coquillet) [37], *B. oleae* (Rossi) [38], and *B. correcta* (Bezzi) [39]. In most tephritid fruit flies, males are thought to produce sex pheromones to attract females [40]. However, there are several notable exceptions. For example, 1,7-dioxaspiro[5,5]undecane has been described as a female-produced pheromone of *B. oleae* [38], although later studies reported on this compound in rectal glands of young males of *B. oleae* [41]. While 1,7-dioxaspiro[5.5]undecane has been used extensively for the monitoring and mass trapping of *B. oleae* [42], the sex specific olfactory cues of *B. oleae* are driven by synergistic actions of a number of compounds that are not yet fully understood [4,43]. In *Z. cucurbitae*, females are attracted by male rectal gland secretions containing three aliphatic amides, two pyrazines, and an aromatic acid [44], while males are attracted by headspace constituents containing 2,8-dialkyl-1,7-dioxaspiro[5.5]undecanes and *N-*(3-methylbutyl)acetamide [45]. Similarly, males of *B. dorsalis* produce two phenols and an aliphatic cyclic alcohol in their rectal glands that show pheromonal activity towards females [46,47], while females emit several spiroacetals that attract males [45].

The chemical profiles of the *B. frauenfeldi* volatile compounds are unknown. Given the central role of chemical-mediated sexual communication in the reproductive biology of tephritid fruit flies [4,48], and the potential application of volatiles as attractants, the present study analysed the rectal gland extracts and headspace collections from both males and females of *B. frauenfeldi* using gas chromatography-mass spectrometry (GC-MS). In order to identify electrophysiologically active components present in the emissions of each sex, we used gas chromatography-electroantennogram detection (GC-EAD) and gas chromatography-electropalpogram detection (GC-EPD) to test the responses of male and female antennae and maxillary palps to the emissions of male and female rectal glands. The rectal gland contents were tested for attraction of the opposite and same sex using y-maze olfactometers [49,50,51].

## 2. Methods and Materials

### 2.1. Insects

Pupae of *B. frauenfeldi* (9 generations from wild) were obtained from the Queensland Department of Agriculture and Fisheries (Cairns, Australia). Approximately 500 pupae were allowed to emerge in a 47.5 × 47.5 × 47.5 cm fine mesh cage (Megaview Bugdorm 4S4545, Taiwan) in a controlled environment room (25 ± 0.5 °C, 65 ± 5% relative humidity) and with light:dusk:dark:dawn 11.5:0.5:11.5:0.5 h photoperiod (these conditions were maintained for all rearing and experiments). The adult flies were fed sugar and yeast hydrolysate (MP Biomedicals LLC) and were provided water through a soaked sponge. The flies were reared through one generation at Macquarie University, Sydney, using a standard carrot larval diet [52], following methods described by Pérez et al. [53]. The flies were separated by sex within three days after emergence and transferred to 12.5 L clear plastic cages (180 flies per cage). No mating was observed before separating the flies. The flies used for all the experiments were 13–18 days old.

### 2.2. Gland Extraction

The flies were killed by chilling them on dry ice. The rectal glands were extracted under a stereomicroscope by gently pressing the abdomen and pulling the gland out with fine forceps. The glands were carefully placed in a 1.1 mL tear-drop vial in dry ice. Once 10 glands were collected, the vials were removed from the dry ice and 100 μL of *n*-hexane (HPLC grade, Sigma-Aldrich, St. Louis, MO, USA) was added. Vials containing *n*-hexane and glands were left to stand at room temperature for 10 min, and then the extracts were transferred to a new vial, labelled, and stored at −20 °C until analysed [54]. Ten replicates of 10 glands were collected for each sex.

### 2.3. Headspace Collections

Based on our preliminary observations, *B. frauenfeldi* appeared to mate at any time during the day, with the mating peak at noon. Ten males or females were separately placed into a cylindrical glass chamber (150 mm long and 40 mm ID) 30 min before the mating peak. A charcoal-filtered air stream at a flow rate of 0.5 L/min (air pulling system) was drawn over the flies for 1 h. The released volatiles were adsorbed onto traps of 50 mg of Tenax-GR Mesh 60/80 adsorbent (Scientific Instrument Services, Inc, Palmer, MA, USA) packed into 6 × 50 mm glass tubes and fitted with glass wool plugs. The volatiles were eluted with 1 mL of *n*-hexane. The samples were stored at −20 °C until analysed. Seven replicates were collected for each sex. To distinguish any possible contaminants, an air control sample comprising an empty glass chamber was run and analysed along with each volatile collection.

Before each headspace collection, the glass chambers were washed with 5% Extran aqueous solution, rinsed with hot tap water, and heated at 200 °C for 18 h. The tenax traps were thermally conditioned at 200 °C for three hours under a 75 mL/min nitrogen stream. The activated charcoal filters were conditioned by heating them at 200 °C for 18 h prior to each headspace collection [55].

### 2.4. GC-MS Analysis

Mass spectra were recorded on a Shimadzu GCMS-TQ8040 instrument (Kyoto, Japan), using a capillary column with 5% diphenyl/95% dimethyl polysiloxane as the stationary phase (SH-Rtx-5MS, 30 m × 0.25 mm ID × 0.25 μm film thickness, Shimadzu, Japan) and helium (99.999%) (ultra-high purity, BOC, Sydney, Australia) as a carrier gas with a constant flow of 1 mL/min. A 1 μL sample was injected in the splitless mode. The injector temperature was set at 270 °C. The temperature program was 40 °C (1 min) to 250 °C (3 min) at a rate of 10 °C/min. The interphase and ion source temperatures were set at 290 °C and 200 °C, respectively. Mass spectra were recorded in electron impact mode (70 eV), scanning from 40 to 500 *m*/*z*. A peak was considered of interest if it was not present in the air control samples. Compounds including esters, amides, and spiroacetals were identified through comparison with retention times and fragmentation patterns of authentic samples, with the exception of compound **4** which was tentatively identified based on literature mass spectral fragmentation patterns [56,57]. Ethyl caprate (**5**), methyl laurate (**6**), ethyl laurate (**7**), ethyl tridecanaote (**8**), propyl laurate (**9**), methyl myristate (**10**), myristic acid (**11**), ethyl myristoleate (**12**), ethyl myristate (**13**), methyl palmitoleate (**14**), methyl palmitate (**15**), palmitoleic acid (**16**), palmitic acid (**17**), ethyl palmitate (**19**), methyl elaidate (**20**), and ethyl oleate (**21**) were purchased from Sigma-Aldrich (Castle Hill, Australia), Alfa-Aesar (United Kingdom), Nu-Chek-Prep, and INC (Minneapolis, USA). Ethyl palmitoleate (**18**), ethyl elaidate (**22**), *N-*(2-methylbutyl)acetamide (**1**), *N-*(3-methylbutyl)acetamide (**2**), and (*E*,*E*)-2,8-dimethyl-1,7-dioxaspiro[5,5]undecane (**3**) were synthesised (see Supplementary Materials for synthesis details).

### 2.5. Electrophysiological Assays

Gas chromatography-electroantennographic detection (GC-EAD) and gas chromatography-electropalpogram detection (GC-EPD) were carried out to identify the antennal- or palpal-active components from male and female rectal gland extracts. The system consisted of a gas chromatography flame ionization detector (Agilent 7890B, CA, USA) coupled to an electroantennogram (Syntech, Hilversum, The Netherlands). The GC was equipped with a polar capillary column with (35%-phenyl)-methylpolysiloxane as the stationary phase (Agilent HP-5, 30 m × 0.32 mm ID × 0.25 μm film thickness). The carrier gas was hydrogen (99.999% pure) supplied by a generator (MGG-2500-220 Parker Balston, NY, USA) with a constant flow of 2.5 mL/min. The initial temperature was set at 50 °C (1 min) then increased to 250 °C (3 min) at a rate of 10 °C/min. The injector and detector temperatures were set at 270 °C and 290 °C, respectively. The effluent of the column was mixed with 30 mL/min make-up nitrogen gas and split in a ratio of 1 (FID) to 1.5 (EAD) through a heated transfer line (Syntech, TC-02, Syntech, Hilversum, The Netherlands) and kept at 200 °C.

A female or male *B. frauenfeldi* head was carefully severed and a borosilicate glass capillary electrode filled with electrically conductive gel (Spectra 360) was attached onto the back of the head. The tip of the antenna or maxillary palp was inserted into the tip of the recording glass capillary electrode filled with phosphate-buffered saline (PBS). The mounted heads were under charcoal filtered and humidified air flow (400 mL/min) controlled by a flow controller (Syntech Stimulus Controller CS-55, Syntech, Hilversum, The Netherlands) and were subjected to each stimulus. The electrophysiological responses were captured and processed by a data acquisition controller (IDAC-4, Syntech, Hilversum, The Netherlands). Before the injection of the sample into the airstream, the antenna/palp was stimulated with 1-hexanol to check its sensitivity. EAD/EPD signals were analysed using GC-EAD 2014 software version 1.2.5. Nine successful GC-EAD/EPD recordings were obtained for each sex. The electrophysiological responses of male and female antennae and palps to the conspecific opposite and same sex rectal gland extracts were recorded. A response was considered genuine if it was present in at least six out of the nine replicates collected. The identity of the compounds eliciting an electrophysiological response was confirmed by comparing the retention times with that of the GC-MS chromatograms using the same column and method as for the GC(FID)-EAD experiments.

### 2.6. Y-maze Bioassays

The response of sexually mature (13–18 days old) *B. frauenfeldi* males and females toward rectal gland contents of the same and opposite sex was evaluated using Y-maze olfactometers. The system consisted of a clear Plexiglas Y shape tube with one central arm (6.5 cm × 4.5 cm × 5 cm) in which the release chamber (5cm × 5cm × 5cm) was located, and two upwind lateral arms (12.5 cm × 4.5 cm × 5 cm), each of which was connected to a rectangular chamber (7.5 cm × 5 cm × 5 cm) (see Supplementary Materials, Figure S1). The Y-maze olfactometer was positioned horizontally on a white table and a humidified and charcoal-filtered air stream was passed through the Y-maze at a flow rate of 140 ± 5 mL/min. The stimulus cartridge was prepared by crushing 15 rectal glands (male or female) on a 1 cm^2^ filter paper (Advantec, Tokyo, Japan) inserted in a glass Pasteur pipette (145 mm long). The control cartridge was prepared using 1 cm^2^ filter paper inserted in the same type of glass Pasteur pipette. One cartridge of each type was fitted to one of the Y-maze upwind arms using a Tygon tube (Tygon® formula E-3603, Sigma-Aldrich, St. Louis, MO, USA). An individual fly was placed in the release chamber to acclimatise for 30 min before each experiment. The experiment was carried out at noon in a controlled environment room, under the same conditions the flies were kept. Each trial lasted 30 min. Once the two cartridges (stimulus and control) were connected to the upwind arms, the system was allowed to equilibrate for two minutes and then the barriers of the two upwind arms and the release chamber were removed. A choice was recorded when the fly reached one of the two upwind arms and stayed there for at least one minute. Flies that did not make any choice, i.e. remained in the release chamber, did not reach one of the two upwind arms, or did not stay there for one minute, were excluded. For each treatment, 45–50 replicates from responsive flies were carried out over multiple days with no more than 15 replicates on any day. The position (left or right) of the stimulus and the control was reversed for every trial to minimise the positional effects. The flies used in this experiment were obtained from multiple batches (two per week) and each fly was tested only once. Freshly dissected rectal glands were used each day. Before each replicate, the Y-maze olfactometer was washed with 5% Extran aqueous solution, rinsed with hot tap water, and air-dried. To compare the number of flies choosing the stimulus over the control, a binomial test with the probability level of *p* < 0.05 was used.

## 3. Results

**Figure 1:**
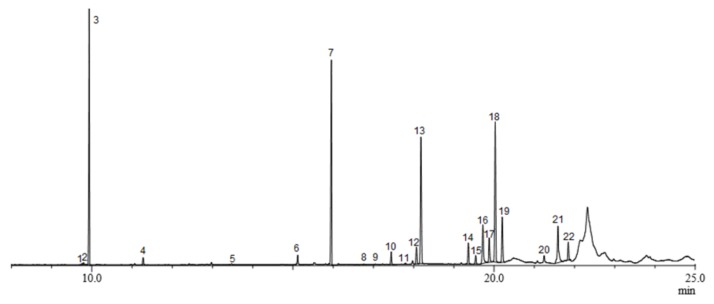
Gas chromatogram of the rectal gland extract of *B. frauenfeldi* females. The numbered peaks indicate detected compounds: *N-*(2-methylbutyl)acetamide (**1**), *N-*(3-methylbutyl)acetamide (**2**), (*E*,*E*)-2,8-dimethyl-1,7-dioxaspiro[5,5]undecane (**3**), (*E*,*E*)-2-ethyl-8-methyl-1,7-dioxaspiro[5.5]undecane (**4**), ethyl caprate (**5**), methyl laurate (**6**), ethyl laurate (**7**), ethyl tridecanaote (**8**), propyl laurate (**9**), methyl myristate (**10**), myristic acid (**11**), ethyl myristoleate (**12**), ethyl myristate (**13**), methyl palmitoleate (**14**), methyl palmitate (**15**), palmitoleic acid (**16**), palmitic acid (**17**), ethyl palmitoleate (**18**), ethyl palmitate (**19**), methyl elaidate (**20**), ethyl oleate (**21**), and ethyl elaidate (**22**).

The GC-MS results showed that male and female *B. frauenfeldi* have different rectal gland and volatile emission compositions. Females produced a more complex blend than did males (Figure 1). A total of twenty-two compounds were identified including amides (**1** and **2**), spiroacetals (**3** and **4**), esters (**5**–**10**, **12**–**15**, **18**–**21**, and **22**), and fatty acids (**11**, **16,** and **17**). The identities of the twenty-one compounds were confirmed by comparison with GC retention times and mass fragmentation patterns of authentic samples.

Of the twenty-two compounds that were identified in female *B. frauenfeldi*, nine compounds were detected in rectal gland extracts and headspace samples including *N-*(3-methylbutyl)acetamide (**2**), (*E*,*E*)-2,8-dimethyl-1,7-dioxaspiro[5.5]undecane (**3**), (*E*,*E*)-2-ethyl-8-methyl-1,7-dioxaspiro[5.5]undecane (**4**), methyl laurate (**6**), ethyl laurate (**7**), methyl myristate (**10**), ethyl myristoleate (**12**), ethyl myristate (**13**), and ethyl palmitoleate (**18**) (Table 1). The main compounds present in female gland extracts and headspace samples were (*E*,*E*)-2,8-dimethyl-1,7-dioxaspiro[5.5]undecane (**3**) and ethyl laureate (**7**), although they were found in higher proportions in the headspace samples (Table 1). 

**Figure 2:**
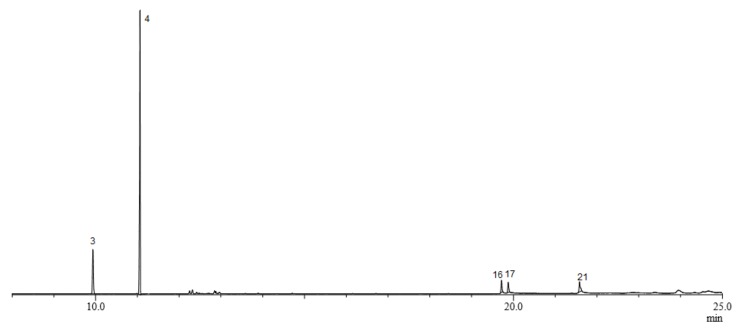
Typical Gas chromatogram of rectal gland extract of *B. frauenfeldi* males. The numbered peaks indicate detected compounds: (*E*,*E*)-2,8-dimethyl-1,7-dioxaspiro[5.5]undecane (**3**), (*E*,*E*)-2-ethyl-8-methyl-1,7-dioxaspiro[5.5]undecane (**4**), palmitoleic acid (**16**), palmitic acid (**17**), and ethyl oleate (**21**).

The GC-MS analysis of male rectal gland extracts showed a blend of six compounds (Figure 2), being (*E*,*E*)-2-ethyl-8-methyl-1,7-dioxaspiro[5.5]undecane (**4**) as the main compound and (*E*,*E*)-2,8-dimethyl-1,7-dioxaspiro[5.5]undecane (**3**) as the second major compound, representing 70% and 17% of the blend, respectively. Male headspace samples showed only (*E*,*E*)-2-ethyl-8-methyl-1,7-dioxaspiro[5.5]undecane and (*E*,*E*)-2,8-dimethyl-1,7-dioxaspiro[5.5]undecane, in a similar ratio to that detected in the male rectal gland samples.

### 3.1. Electrophysiological Responses

**Figure 3:**
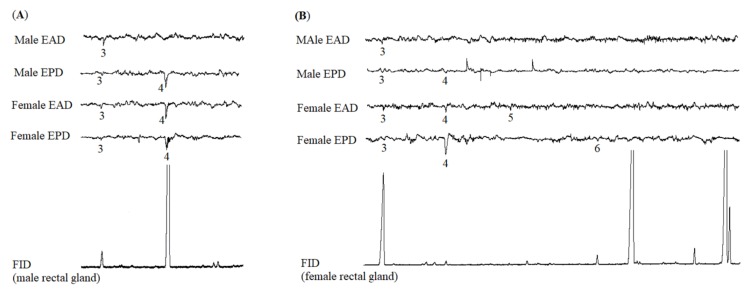
Flame ionization detector (FID) response and electrophysiological responses of antennae (EAD) and maxillary palps (EPD) using *Bactrocera frauenfeldi* males and females to (**A**) rectal gland extracts from conspecific males and (**B**) rectal gland extracts from conspecific females. The numbered peaks indicate EAD- and EPD-active compounds: (*E*,*E*)-2,8-dimethyl-1,7-dioxaspiro[5.5]undecane (**3**), (*E*,*E*)-2-ethyl-8-methyl-1,7-dioxaspiro[5.5]undecane (**4**), ethyl caprate (**5**), and methyl laurate (**6**).

The analysis by GC-EAD of the male rectal gland samples showed that female and male antennae responded consistently to the main spiroacetal, (*E*,*E*)-2,8-dimethyl-1,7-dioxaspiro[5.5]undecane (**3**). The female antennae also responded to (*E*,*E*)-2-ethyl-8-methyl-1,7-dioxaspiro[5.5]undecane (**4**) from the male rectal gland, while the male antennae did not. The female and male palps responded to (*E*,*E*)-2,8-dimethyl-1,7-dioxaspiro[5.5]undecane (**3**) and (*E*,*E*)-2-ethyl-8-methyl-1,7-dioxaspiro[5.5]undecane (**4**) (Figure 3A).

In contrast, GC-EAD analysis of female rectal gland samples revealed that (*E*,*E*)-2,8-dimethyl-1,7-dioxaspiro[5.5]undecane (**3**) elicited an antennal response in both males and females. The female antennae responded to two more compounds from the female rectal gland including (*E*,*E*)-2-ethyl-8-methyl-1,7-dioxaspiro[5.5]undecane (**4**) and ethyl caprate (**5**). GC-EPD analysis of the female rectal gland samples showed that the female and male maxillary palps responded to (*E*,*E*)-2,8-dimethyl-1,7-dioxaspiro[5.5]undecane (**3**) and (*E*,*E*)-2-ethyl-8-methyl-1,7-dioxaspiro[5.5]undecane (**4**). Methyl laurate (**6**) was only detected by the female palps (Figure 3B).

### 3.2. Y-maze Bioassays

**Figure 4:**
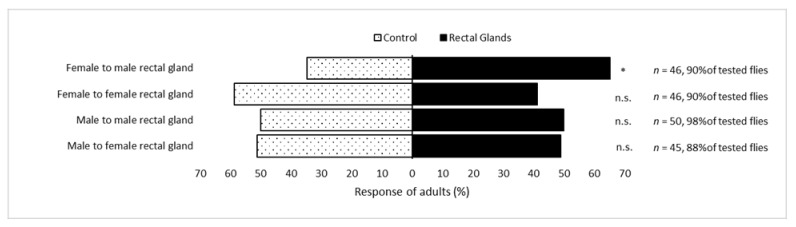
Response of sexually mature virgin *Bactrocera frauenfeldi* males and females to rectal gland volatiles of the same and opposite sex, vs. control (clean filter paper) in Y-maze bioassays. * significantly different at 0.01 level, ns not significantly different, n total number of responded flies.

Sexually mature *B. frauenfeldi* females significantly preferred the upwind arm containing male rectal glands over the control (*P* = 0.01). In contrast, *B. frauenfeldi* males did not show a significant preference for female rectal glands over the control (*P* = 0.1). Neither females nor males exhibited significant preferences when the rectal gland content of the same sex was presented (*P* = 0.06 and *P* = 0.1, respectively) (Figure 4).

## 4. Discussion

We report here the first identification, electrophysiological detection, and behavioural evaluation of rectal gland volatiles produced by *B. frauenfeldi* males and females. Our data show that females of this species produced and emitted a greater diversity of compounds than males. The compounds included aliphatic amides, spiroacetals, and saturated and unsaturated acids and esters. The two amides, *N*-(2-methylbutyl)acetamide (**1**) and *N-*(3-methylbutyl)acetamide (**2**), found in this study have been previously reported in rectal glands of other species, including *B. tryoni* [31,58]*, Z. cucumis* (French) [34], *B. dorsalis* (Hendel), and *Z. cucurbitae* (Coquillett) [45]. *N-*(3-Methylbutyl)acetamide is one of the major components in the rectal glands of *B. tryoni* males. The two spiroacetals that were found in *B. frauenfeldi* have also been reported in other *Bactrocera* species, and closely related *Zeugodacus*. For instance, *EE*, *EZ,* and *ZZ* isomers of 2,8-dimethyl-1,7-dioxaspiro[5.5]undecane have been reported in the rectal glands of *Z. cucumis* [33]. (*E*,*E*)-2,8-Dimethyl-1,7-dioxaspiro[5.5]undecane (**3**) have been also found in the rectal glands of *B. dorsalis*, *B. nigrotibialis* (Perkins), *B. albistrigata* (Meijere), *B. jarvisi* (Tryon), *B. kirki* (Froggatt), *B. kraussi* (Hardy), *B. musae* (Tryon), and *B. tryoni* [33,34,45,58,59,60,61]. This compound was also found in both sexes of *B. frauenfeldi*, although it was less abundant in males. The most abundant compound in males was (*E*,*E*)-2-ethyl-8-methyl-1,7-dioxaspiro[5.5]undecane. The spiroacetal (*E*,*E*)-2-ethyl-8-methyl-1,7-dioxaspiro[5.5]undecane has been previously reported in the rectal glands of male *B. nigrotibialis* (Perkins), *B. halfordiae* (Tryon), *B. dorsalis* (Hendel), *B. kirki* (Froggatt), *B. latifrons* (Hendel), and *B. occipitalis* (Bezzi) and in female *B. tryoni* (Froggatt) and *B. musae* [4,58,61,62,63]. In addition to the compounds found in males, the female rectal gland and headspace extracts included saturated and unsaturated acids and esters. The compounds, ethyl caprate (**5**), methyl laurate (**6**), ethyl laurate (**7**), ethyl tridecanaote (**8**), propyl laurate (**9**), methyl myristate (**10**), myristic acid (**11**), ethyl myristoleate (**12**), ethyl myristate (**13**), methyl palmitoleate (**14**), methyl palmitate (**15**), ethyl palmitoleate (**18**), ethyl palmitate (**19**), methyl elaidate (**20**), and ethyl elaidate (**22**) were female-specific. Nine of the saturated and unsaturated esters in *B. frauenfeldi* have also been reported in *B. oleae*, with ethyl myristate and ethyl palmitate as the major compounds in both species [49]. *B. musae* rectal glands were also reported to contain twelve of the esters found in female *B. frauenfeldi* with ethyl laurate, ethyl myristate, and ethyl palmitate as the most abundant components [61]. Ethyl caprate, which was EAG active for females of *B. frauenfeldi*, has been found to attract both males and females of *B. oleae* [49]. The other electrophysiologically active ester in this study, methyl laurate, has not been reported as an attractant in any other species, but the similar saturated methyl ester, methyl palmitate, has been reported to attract both male and female *B. oleae* [49]. Many of the saturated/unsaturated acids and esters that we found in *B. frauenfeldi* females are also common in females of other *Bactrocera* species, although ratios vary amongst species.

The differences between the composition of rectal glands and volatile emissions observed in this study are not likely due to sampling and/or sensitivity of the instrument because allowing for those factors, the ratios in the rectal gland samples would be matched in the headspace collections. This is not the case. In female rectal glands compounds **5** and **6** have similar relative abundance (< 1%) but only compound **6** was detected in the headspace samples. Similarly, compounds **16** and **19** were more abundant than compound **6** in female rectal glands (both > 5%) but were not detected in headspace samples. For the compounds with longer chain length, and hence lower volatility including the C14, C16, and C18 esters, compared to the C12 ester, it is likely that the relative abundance of these compounds is lower in headspace than rectal glands. The differences between the composition of rectal glands and volatile emissions could be due to the disproportionate release of some compounds.

The proportion of compounds in a blend can be critical for triggering behavioural responses, and the differences might be responsible for sex-specific behavioural responses. For example, in closely related moth species, pheromone blends often contain the same compounds but in different ratios [64,65]. In the present study we found sex differences in the proportion of some compounds. For instance, the spiroacetals **3** and **4** are present in very different ratios in the rectal gland of males and females; 19:81 in males vs. 99:1 in females. If the presence of the compounds was solely responsible for behavioural response, females should have had a preference for female glands in Y-maze bioassays. The fact that this was not the case suggests natural proportions of the compounds may be important in triggering sex-specific behavioural responses.

Male production of sex-attracting pheromones has been reported in diverse tephritid fruit flies [25,34,66]. For example, the male Caribbean fruit fly, *Anastrepha sunspensa* (Loew), releases volatile pheromone components including (*Z*)-non-3-en-1-ol and (3*Z*,6*Z*)-nona-3,6-dien-1-ol, anastrephin, and epianastrephin, which attract female conspecifics [25,66]. The male Mediterranean fruit fly, *Ceratitis capitata* (Wiedemann), releases a cyclic imine, l-pyrroline, that is highly attractive to virgin females [34,67]. In *B. tryoni*, male glandular blends include the amides, *N*-3-methylbutylpropanamide, *N*-3-methylbutylacetamide, *N*-(3-methylbutyl)-2-methylpropanamide, *N*-2-methylbutylpropanamide, *N*-2-methylbutylacetamide, and *N*-(2-methylbutyl)-2-methylpropanamide, which are thought to function as short-range stimulants for females [31,34]. Our Y-maze olfactometer results showed that female *B. frauenfeldi* are attracted to male gland odour, whereas males did not exhibit a significant preference for male or female gland odours during the period of peak mating activity. This suggests that a specific compound or specific compounds produced in the rectal gland of *B. frauenfeldi* males elicits attraction of females. Our GC-EAD/EPD results showed that (*E*,*E*)-2,8-dimethyl-1,7-dioxaspiro[5.5]undecane elicits antennal and palpal responses in both males and females. Although (*E*,*E*)-2-ethyl-8-methyl-1,7-dioxaspiro[5.5]undecane elicits an antennal response only in females, it was detected by palps in both males and females. Our findings suggest that in *B. frauenfeldi* males are the main sex pheromone producer and (*E*,*E*)-2,8-dimethyl-1,7-dioxaspiro[5.5]undecane and (*E*,*E*)-2-ethyl-8-methyl-1,7-dioxaspiro[5.5]undecane are sex pheromone candidates. This chemical information about the sexual communication of *B. frauenfeldi* provides a valuable foundation for the synthesis and development of new nature-inspired attractants for the control of this pest species.

## Figures and Tables

**Table 1:** Percentage of compounds identified in chemical profiles for *B. frauenfeldi*. RT = retention time, KI = Kovats index, ND = not detected.

Compound	Females		Males		RT	KI	Diagnostic Ions *m*/*z* (%)
	Headspace (%)	Rectal Gland (%)	Headspace (%)	Rectal Gland (%)			
*N-*(2-Methyl-butyl)acetamide (**1**)	ND	<1	ND	ND	9.7	1133	129 (M^+^, 5.2), 100 (62.2), 73 (β-cleavage/H rearrangement, 76.4), 72 (M – C_4_H_9_, 100), 60 (CH_3_C(OH)NH^+^, 54.8)
*N-*(3-Methylbutyl)acetamide (**2**)	<1	<1	ND	ND	9.8	1137	129 (M^+^, 6.6), 114 (18.2), 86 (28.4), 73 (β-cleavage/H rearrangement, 100), 72 (M – C_4_H_9_, 74.4), 60 (CH_3_C(OH)NH^+^, 32.6)
(*E*,*E*)-2,8-Dimethyl-1,7-dioxaspiro[5.5]undecane (**3**)	53.9	20.1	24.6	16.6	9.9	1147	184 (M^+^, 9.7), 169 (2.1), 140 (17.8), 125 (9.7), 115 (CH_3_(C_5_H_7_O)=OH^+^, 98.1), 112 (CH_3_(C_5_H_7_O)=CH_2_, 100), 97 (75.4), 69 (33.3), 55 (31.2)
(*E*,*E*)-2-Ethyl-8-methyl-1,7-dioxaspiro[5.5]undecane (**4**)	5.7	<1	75.4	70.3	11.3	1237	198 (M^+^, 10.7), 169 (14.1), 140 (17.5), 129 (CH_3_CH_2_(C_5_H_7_O)=OH^+^, 52), 126 (CH_3_CH_2_(C_5_H_7_O)=CH_2_, 40.1), 115 CH_3_(C_5_H_7_O)=OH^+^, 94.2), 112 (CH_3_(C_5_H_7_O)=CH_2_, 100), 97 (66.5), 69 (43.5), 55 (49.1)
Ethyl caprate (**5**)	ND	<1	ND	ND	13.5	1396	200 (M^+^, 1.7), 171 (4.2), 157 (19.5), 155 (M – OC_2_H_5_, 15.9), 115 (9.7), 101 (44.7), 88 (100), 73 (COOC_2_H_5_, 23.6), 70 (27.6)
Methyl laurate (**6**)	2.1	<1	ND	ND	15.1	1524	214 (M^+^, 3.7), 183 (M – OCH_3_, 7.8), 171 (14.6), 143 (18.2), 87 (60), 74 (100), 59 (COOCH_3_, 8.4), 55 (22.8)
Ethyl laurate (**7**)	30.3	18.9	ND	ND	15.9	1595	228 (M^+^, 4.3), 199 (4.7), 183 (M – OC_2_H_5_, 11.6), 157 (18.2), 101 (52.9), 88 (100), 73 (COOC_2_H_5_, 20.9), 70 (25.8), 61 (13.6), 55 (21.3)
Ethyl tridecanaote (**8**)	ND	<1	ND	ND	16.8	1667	242 (M^+^, 4.5), 213 (11.9), 199 (15.6), 197 (M – OC_2_H_5_, 2.3), 157 (31.7), 101 (60.9), 88 (100), 73 (COOC_2_H_5_, 5.8), 57 (25.9), 55 (24.4)
Propyl laurate (**9**)	ND	<1	ND	ND	17.1	1691	242 (M^+^, 1.6), 201 (40.4), 199 (1.1), 183 (M – OC_3_H_7_, 36.5), 115 (26.7), 102 (29.7), 87 (COOC_3_H_7_, 11.2), 61 (100), 60 (34), 55 (30.4)
Methyl myristate (**10**)	<1	1.4	ND	ND	17.4	1727	242 (M^+^, 6.6), 211 (M – OCH_3_, 6.3), 199 (16.2), 143 (25.6), 87 (64.4), 74 (100), 59 (COOCH_3_, 7.8), 55 (23.4)
Myristic acid (**11**)	ND	<1	ND	ND	17.8	1759	228 (M^+^, 19.8), 185 (44.6), 171 (26.6), 143 (25.2), 129 (67.6), 115 (24.5), 97 (22.1), 87 (33.1), 85 (21.2), 83 (25.7), 73 (100), 69 (39.3), 60 (CH_3_COOH, 90.6), 57 (68), 55 (64)
Ethyl myristoleate (**12**)	2.6	1.9	ND	ND	18.1	1785	254 (M^+^, 4.1), 209 (M – OC_2_H_5_, 13.9), 208 (M – C_2_H_5_OH, 14.9), 166 (28.8), 124 (23.7), 88 (46.3), 73 (COOC_2_H_5_, 16.6), 69 (52.1), 55 (100)
Ethyl myristate (**13**)	1.9	14.6	ND	ND	18.2	1795	256 (M^+^, 7.1), 213 (13.8), 211 (M – OC_2_H_5_, 8.16), 157 (21.9), 101 (53.8), 88 (100), 73 (COOC_2_H_5_, 17.8), 70 (22.1), 55 (20.1)
Methyl palmitoleate (**14**)	ND	2.5	ND	ND	19.3	1909	268 (M^+^, 5.1), 237 (M – OCH_3_, 14.2), 236 (M – CH_3_OH, 18.5), 194 (17.9), 152 (24.1), 96 (51.3), 74 (52.3), 59 (COOCH_3_, 17.1), 55 (100)
Methyl palmitate (**15**)	ND	<1	ND	ND	19.5	1928	270 (M^+^, 12.5), 227 (14.8), 143 (23.6), 87 (68.2), 74 (100), 69 (12.5), 59 (COOCH_3_, 7.2), 55 (24.8)
Palmitoleic acid (**16**)	ND	5.5	ND	4.4	19.7	1825	254 (M^+^, 2.2), 236 (13.6), 152 (9.2), 111 (23.8), 98 (33.8), 97 (50.3), 96 (35.2), 83 (56.4), 73 (15.3), 69 (73.7), 60 (CH_3_COOH, 10), 57 (24.8), 55 (100)
Palmitic acid (**17**)	ND	3.1	ND	3.9	19.9	1962	256 (M^+^, 38.1), 227 (9.9), 213 (M – COOH, 31.3), 185 (26.9), 157 (31.4), 129 (61.8), 115 (26.5), 97 (33.2), 87 (36.7), 85 (37), 83 (39), 73 (100), 69 (45.9), 60 (CH_3_COOH, 84.8), 57 (88.9), 55 (75.4)
Ethyl palmitoleate (**18**)	2.5	16.1	ND	ND	20.0	1977	282 (M^+^, 2.9), 237 (M – OC_2_H_5_, 19.1), 236 (M – C_2_H_5_OH, 21.3), 194 (23.2), 152 (28.6), 88 (57.3), 73 (COOC_2_H_5_, 16.8), 69 (68.7), 55 (100)
Ethyl palmitate (**19**)	ND	5.3	ND	ND	20.2	1995	284 (M^+^, 11.2), 255 (4.1), 241 (13.2), 239 (M – OC_2_H_5_, 7.5), 157 (21.3), 101 (57.5), 88 (100), 73 (COOC_2_H_5_, 16.1)
Methyl elaidate (**20**)	ND	<1	ND	ND	21.2	2102	296 (M^+^, 5.3), 265 (M – OCH_3_, 17.8), 264 (26.7), 222 (16.9), 152 (13.6), 97 (62.2), 74 (47.5), 69 (66.2), 55 (100)
Ethyl oleate (**21**)	ND	6.4	ND	4.8	21.6	2144	310 (M^+^, 1.2), 265 (M – OC_2_H_5_, 8.8), 264 (M – C_2_H_5_OH, 16.9), 222 (5.4), 123 (13.6), 110 (22.8), 97 (59.7), 88 (54.1), 83 (62.9), 73 (COOC_2_H_5_, 15.1), 69 (72.1), 55 (100)
Ethyl elaidate (**22**)	ND	1.9	ND	ND	21.8	2172	310 (M^+^, 7.9), 265 (M – OC_2_H_5_, 24.5), 264 (M – C_2_H_5_OH, 31.7), 222 (22.1), 180 (20.4), 110 (31.4), 97 (65.5), 88 (57.9), 83 (63.4), 73 (COOC_2_H_5_, 15.2), 69 (68.4), 55 (100)

## Supplementary Materials

The supplementary materials are available online. Synthesis of *N*-(2-methylbutyl)acetamide, *N*-(3-methylbutyl)acetamide, 2,8-dimethyl-1,7-dioxaspiro[5.5]undecane, ethyl palmitoleate, and ethyl elaidate. Figure S1. Y-maze apparatus used in this study.
