# Supplementary material for: Attraction and Electrophysiological Response to Identified Rectal Gland Volatiles in Bactrocera frauenfeldi (Schiner)
Source: Molecules. 2020 Mar 11;25(6):1275. doi: 10.3390/molecules25061275 (PMC7143976; doi:10.3390/molecules25061275)
Supplement: Supplementary file 1 [file molecules-25-01275-s001.pdf]

Supplementary Material for

Attraction and Electrophysiological Response to Identified Rectal Gland Volatiles in *Bactrocera frauenfeldi* (Schiner)

Saeedeh Noushini[1,3*], Jeanneth Perez[2,3], Soo Jean Park[1,3], Danielle Holgate[1], Vivian Mendez Alvarez[2,3], Ian Jamie[1,3], Joanne Jamie[1, 3], Phillip Taylor[2,3]

[1] *Department of Molecular Sciences, Macquarie University, Sydney, NSW 2109, Australia.*

[2] *Applied BioSciences, Macquarie University, Sydney, NSW 2109, Australia.*

[3] *Australian Research Council Industrial Transformation Training Centre for Fruit Fly Biosecurity Innovation, Macquarie University, Sydney, NSW 2109, Australia.*

Corresponding author:
Saeedeh Noushini, Department of Molecular Sciences, Macquarie University, Sydney, NSW 2109, Australia
E-mail**:** sally.noushini@mq.edu.au
ORCID: 0000-0001-5558-1656

General Procedures for Synthesis

All reagents were purchased from Sigma-Aldrich and used without further purification. All solvents were anhydrous or analytical grade (Sigma-Aldrich) and used without further purification. The reaction progress was monitored by GC-MS (using the procedure given in the main text). Solvents were removed under reduced pressure using a Büchi Rotavapor R-200 and Büchi B-490 heating bath set to 40 °C. Mixtures were further dried under high vacuum using an Alcatel Pascal 2005SD vacuum pump. NMR spectra were recorded on a Bruker AVANCE-400 instrument (^{1}H NMR: 400 MHz, ^{13}C NMR: 101 MHz) using $CDCl_3$ and C_6D_6. The ^{1}H NMR chemical shifts were referenced to the residual protonated solvent peaks at δH 7.26 for chloroform-d and 7.15 for benzene-d_6. ^{13}C NMR chemical shifts were referenced to the central solvent peaks of bulk solvent at δC 77.16 for chloroform-d and 127.68 for benzene-d_6. *J* values are given in Hz.

Synthesis of *N*-(2-methylbutyl)acetamide (**1**).

The synthesis was conducted using the method of Naik et al. [1]. To a mixture of 2-methylbutylamine (4.4 g, 50 mmol) in water (50 mL) was added acetic anhydride (7.7 g, 75 mmol). The clear reaction mixture was stirred at room temperature for 0.5 hour and the completion of the reaction at this time was determined by GC-MS. The clear reaction mixture was extracted with ethyl acetate (3 × 50 mL). The combined organic layer was washed with 5% aqueous sodium bicarbonate solution (150 mL), dried over sodium sulfate and concentrated under reduced pressure to give the crude product, which was purified by vacuum distillation (150 – 160 °C, 20 mm Hg) to afford *N*-(2-methylbutyl)acetamide (**1**) as a clear liquid (5.6 g, 86% yield). ^{1}H NMR (400 MHz, $CDCl_3$) δ 0.83 (6 H, m, $CHCH_2C\mathbf{H}_3$ and $CH_2C\mathbf{H}_3$), 1.07 (1 H, apparent sep, *J* = 6.6, $C\mathbf{H}CH_3$), 1.29 – 1.53 (2 H, m, $C\mathbf{H}_2CH_3$), 1.93 (3 H, s, $C\mathbf{H}_3CO$), 2.94 – 3.13 (2 H, m, $NC\mathbf{H}_2$), 6.33 (1 H, bs, N**H**). ^{13}C NMR (101 MHz, $CDCl_3$) δ 11.2, 17.1, 23.1, 27.0, 34.8, 45.4, 170.6. GC-MS (EI) *m/z* (%) 129 (M^+, 8), 100 (38), 73 (β-cleavage/H rearrangement, 77.1),72 ($M – C_4H_9$, 100). This compound is known, but spectral data are not available in the literature.

Synthesis of *N*-(3-methylbutyl)acetamide (**2**).

Using a similar reaction, work-up and purification conditions to *N*-(2-methylbutyl)acetamide (**1**) (above), 3-methylbutylamine (5.0 g, 57 mmol) in water (50 mL) was acetylated with acetic anhydride (8.7 g, 86 mmol) to produce *N*-(3-methylbutyl)acetamide (**2**) as a clear liquid (5.4 g, 73% yield). ^{1}H NMR (400 MHz, $CDCl_3$) δ 0.85 (6 H, d, *J* = 6.6, CH(C**H**$_3$)$_2$), 1.33 (2 H, m, C**H**$_2$CH$_3$), 1.56 (1 H, sep, *J* = 6.7, C**H**), 1.92 (3 H, s, C**H**$_3$CO), 3.18 (2 H, m, NC**H**$_2$), 6.21 (1 H, bs, N**H**). ^{13}C NMR (101 MHz, $CDCl_3$) δ 22.4, 23.1, 25.8, 38.0, 38.3, 170.4. GC-MS (EI) *m/z* (%) 129 (M^+, 5), 114 (12), 73 (β-cleavage/H rearrangement, 100), 72 (M – C_4H_9, 73.8). MS data match with those in the literature [2]. NMR data are not available in the literature.

Synthesis of 2,8-dimethyl-1,7-dioxaspiro[5.5]undecane (**3**).

1,10-Undecadien-6-ol. Following the method of Kitching et al. [3], Grignard reaction followed by hydrolysis was conducted to give 1,10-undecadien-6-ol. In brief, a flame-dried argon-flushed two-necked round bottom flask was charged with magnesium (1.8 g, 74 mmol), a single crystal of iodine, and a magnetic stirrer bar, and fitted with a condenser. Dry diethyl ether (60 mL) was added and the suspension was brought to reflux. 5-Bromopent-1-ene (10 g, 67 mmol) in diethyl ether (30 mL) was added dropwise and then the colourless suspension was stirred at reflux for 4 hours. The colourless suspension was cooled to 0 °C and ethyl formate (2.6 g, 34 mmol) was added. The suspension was warmed to room temperature, stirred for 1 hour, then quenched with saturated ammonium chloride and extracted with diethyl ether (3 × 15 mL). The combined organic layers were washed with saturated aqueous brine and dried over magnesium sulfate. After solvent removal by rotary evaporation, the yellow oil was refluxed in 15% aqueous potassium hydroxide solution for 3 hours. The solution was cooled to room temperature and extracted with diethyl ether (3 × 20 mL). Solvent was removed under reduced pressure to give the crude product as a yellow oil, which was purified by distillation (110 – 115 °C, 10 mm Hg) to afford 1,10-undecadien-6-ol as a colourless oil (3.7 g, 60% yield). ^{1}H NMR (400 MHz, $CDCl_3$) δ 5.81 (2 H, ddt, *J* = 17, 10.3, 6.7 Hz, C**H**=), 5.01 (2 H, dq, *J* = 17.1, 1.7 Hz, C**H**$_2$=), 4.91 – 5.01 (2 H, m, C**H**$_2$=), 3.61 (1 H, bs, C**H**OH), 2.00 – 2.13 (4 H, m, C**H**$_2$CH=CH$_2$), 1.26 – 1.61 (9 H, m, including OH). ^{13}C NMR (101 MHz, $CDCl_3$) δ 138.7 (CH=), 114.6 (CH$_2$=), 71.7 (CHOH), 36.9 (CH$_2$), 33.7 (CH$_2$), 24.9 (CH$_2$). GC-MS (EI) *m/z* (%) 84 (12.3), 81 (100), 80 (10),

79 (19.5), 69 (9.2), 68 (9.3), 67 (20.6), 58 (9.5), 57 (30.2), 55 (72.3), 54 (27.7), 53 (9.4), 43 (32.1), 42 (9), 41 (43.8). Spectral data were consistent with the literature [3].

Undeca-1,10-dien-6-one. To a solution of 1,10-undecadien-6-ol (3.99 g, 23.7 mmol) in acetone (10 mL) at -10 °C, freshly prepared Jones reagent (2.7 g of chromium trioxide in 4 mL of sulfuric acid and 12 mL of distilled water) was added dropwise and the reaction monitored by GC-MS. After completion of the reaction (2 hours), the green suspension was filtered through a pad of Celite. The filtrate was washed with saturated aqueous sodium bicarbonate (17 mL), extracted with diethyl ether (4 × 50 mL) and washed with water (50 mL) and saturated aqueous brine (50 mL), then dried over magnesium sulfate. Concentration by rotary evaporation yielded undeca-1,10-dien-6-one as a colourless oil (2.9 g, 75% yield). ^{1}H NMR (400 MHz, $CDCl_3$) δ 5.80 (2 H, ddt, *J* = 17.2, 10.3, 6.7 Hz, C**H**=), 4.87 – 4.97 (4 H, m, C$\mathbf{H_2}$=), 2.33 (4 H, t, *J* = 7.5 Hz, C$\mathbf{H}_2$CO), 1.98 (4 H, m, C$\mathbf{H}_2$CH=CH_2), 1.60 (4 H, quin, *J* = 7.3 Hz, CH_2C$\mathbf{H}_2$$CH_2$). ^{13}C NMR (101 MHz, $CDCl_3$) δ 210.9 (CO), 138.0 (CH=), 115.2 (CH_2=), 41.9 (CH_2), 33.1 (CH_2), 22.8 (CH_2). GC-MS (EI) *m/z* (%) 112 (14.6), 97 (30), 84 (27.8), 83 (10.6), 70 (14.1), 69 (59.5), 58 (48.7), 55 (49.6), 43 (24.5), 41 (100). Spectral data were consistent with the literature [3]

2,8-Dimethyl-1,7-dioxaspiro[5.5]undecane. $Hg(OAc)_2$ (1.9 g, 6.1 mmol) was added to a stirred solution of undeca-1,10-dien-6-one (0.5 g, 3 mmol) in 1% aqueous perchloric acid: tetrahydrofuran (15 mL:15 mL) and the solution was stirred for 15 hours. Benzyltriethylammonium chloride (2.4 g, 10.5 mmol) in 10% aqueous sodium hydroxide (15 mL) and dichloromethane (5 mL) was added followed by sodium borohydride (0.09 g, 2.3 mmol) in 10% aqueous sodium hydroxide (5 mL). The gray suspension was stirred and monitored by GC-MS. After completion of the reaction (20 minutes), the gray suspension was filtered through a pad of Celite, which was then washed with 30 mL of diethyl ether. The aqueous phase was then extracted with diethyl ether (3 × 30 mL) and the combined organic layer (from Celite wash and extraction) were washed with saturated aqueous brine (50 mL) and dried over magnesium sulfate. After solvent removal by rotary evaporation the product was purified by Kugelrohr distillation (bp 110 °C; 30 mm Hg). According to the literature [3] under this condition a mixture of *E*,*E* diastereomer with some *E*,*Z* and no *Z*,*Z* isomer is obtained. These

conformational isomers produced different MS fragmentation patterns that were matched with those in the literature [3].

*(*E,E*)-2,8-Dimethyl-1,7-dioxaspiro[5.5]undecane* (**3**). ^{13}C NMR (101 MHz, C_6D_6) δ 95.75 (CO), 64.8 (CO), 35.33 (CH_2), 32.90 (CH_2), 21.92 (CH_3), 19.03 (CH_2). GC-MS (EI) *m/z* (%) 184 (M^+, 5.6), 169 (1.9), 140 (M−CH_3CHO, 11.6), 125 (8.2), 115 ($CH_3(C_5H_7O)=OH^+$, 92.4), 114 (43.2), 113 (8.6), 112 ($CH_3(C_5H_7O)=CH_2$, 100), 97 (68.4), 84 (15.7), 83 (23.8), 73 (24.8), 71 (18.5), 70 (15.8), 69 (54.9), 58 (18.2), 55 (52.1), 43 (69.4), 42 (35.9), 41 (56.1).

*(*E,Z*)-2,8-Dimethyl-1,7-dioxaspiro[5.5]undecane* (**3**). GC-MS (EI) *m/z* (%) 184 (M^+, 8.1), 115 ($CH_3(C_5H_7O)=OH^+$, 100), 114 (37), 112 ($CH_3(C_5H_7O)=CH_2$, 39.5), 97 (73.1), 83 (11.8), 73 (27.5), 71 (12.4), 69 (59.9), 55 (41.8), 43 (38.6), 42 (24.6), 41 (38.4).

Ethyl Palmitoleate (**18**). A mixture of palmitoleic acid (0.50 g, 1.9 mmol), ethanol (10 mL) and concentrated sulfuric acid (2 drops) was heated to reflux for 1.5 hours. After cooling, diethyl ether (10 mL) and 5% *w/v* aqueous sodium bicarbonate (10 mL) were added to the reaction mixture. The organic layer was separated and washed with 5% *w/v* aqueous sodium bicarbonate (3 × 10 mL) and dried over sodium sulfate. The solvent was removed under reduced pressure, yielding the crude product, which was purified by distillation to give ethyl palmitoleate as a colourless liquid (0.11 g, 19% yield). ^{1}H NMR (400 MHz, $CDCl_3$) δ 5.32 – 5.35 (2 H, m, C**H**=C**H**), 4.11 (2 H, q, *J* = 7.2 Hz, OC$\mathbf{H_2}$$CH_3$), 2.28 (2 H, t, *J* = 7.5 Hz, C$\mathbf{H_2}$COOEt), 1.98 – 2.01 (4 H, m, C$\mathbf{H_2}$CH=CHC$\mathbf{H_2}$), 1.59 – 1.63 (2 H, m, C$\mathbf{H_2}$$CH_2$COOEt), 1.23 – 1.30 (19 H, m, C$\mathbf{H_2}$), 0.88 (3 H, t, *J* = 6.9 Hz, CH_2C$\mathbf{H_3}$). ^{13}C NMR (101 MHz, $CDCl_3$) δ 174.0 (C=O), 130.1 (CH), 129.9 (CH), 60.2 (OCH_2), 34.5 (CH_2), 31.9 (CH_2), 29.87 (CH_2), 29.82 (CH_2), 29.3 (CH_2), 29.26 (CH_2), 29.23 (CH_2), 29.1 (CH_2), 27.36 (CH_2), 27.30 (CH_2), 25.1 (CH_2), 22.8 (CH_2), 14.4 (CH_3), 14.2 (CH_3). GC-MS (EI) *m/z* (%) 282 (M^+, 3.8), 236 (M−OC_2H_5, 14.3), 218 (1.4), 207 (1.4), 194 (15.0), 179 (1.65), 165 (2.8), 152 (14.9), 138 (6.9), 123 (11.9), 101 (31.8), 88 (50.6), 83 (44.9), 69 (64.1), 55 (100), 41 (81.8). Spectral data were not available in the literature.

Ethyl Elaidate (**22**). Using similar conditions to above, elaidic acid (0.45 g, 1.6 mmol), was esterified with ethanol (10 mL) in the presence of concentrated sulfuric acid (2 drops), quenched

with sodium bicarbonate and diethyl ether and purified by distillation to afford ethyl elaidate as a colourless oil (113 mg, 23% yield). ^{1}H NMR (400 MHz, $CDCl_3$) δ 5.36 – 5.28 (2 H, m, C**H**=C**H**), 4.11 (2 H, q, *J* = 7.1 Hz, $OC\mathbf{H_2}CH_3$), 2.27 (2 H, t, *J* = 7.6 Hz, $C\mathbf{H_2}COOEt$), 1.95 – 1.96 (4 H, m, $C\mathbf{H_2}CH{=}CHC\mathbf{H_2}$), 1.57 – 1.60 (2 H, m, $C\mathbf{H_2}CH_2COOEt$), 1.23 – 1.28 (23 H, m, CH_2), 0.87 (3 H, t, *J* = 6.7 Hz, $CH_2CH_2C\mathbf{H_3}$). ^{13}C NMR (101 MHz, $CDCl_3$) δ 174.0 (C=O), 130.6 (CH), 130.4 (CH), 60.3 (OCH_2), 34.5 (CH_2), 32.78 (CH_2), 32.73 (CH_2), 32.0 (CH_2), 29.8 (CH_2), 29.7 (CH_2), 29.6 (CH_2), 29.4 (CH_2), 29.36 (CH_2), 29.30 (CH_2), 29.1 (CH_2), 25.1 (CH_2), 22.8 (CH_2), 14.4 (CH_3), 14.2 (CH_3). GC-MS (EI) *m/z* (%) 310 (M^+, 3.5), 264 ($M^+{-}OC_2H_5$, 16.2), 222 (11.3), 180 (11.2), 155 (7.0), 138 (5.6), 123 (13.5), 111 (20.6), 97 (38.6), 88 (45.6), 83 (49.0), 69 (69.0), 55 (100), 41 (76.4). Spectral data were consistent with the literature [4].

Y-maze apparatus

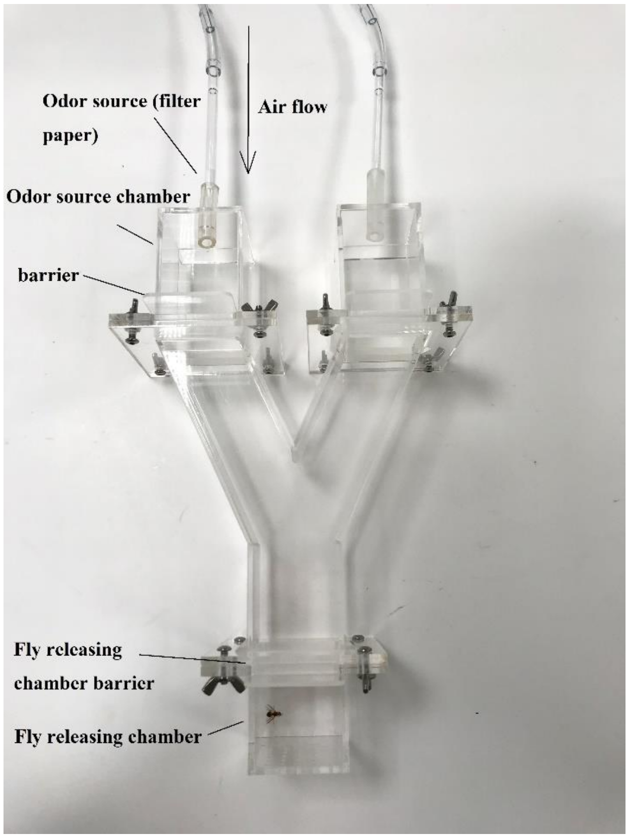

Figure S1. Y-maze apparatus used in this study

Electrophysiology

Responses of antenna (EAD) and maxillary palps (EPD) of female *B. frauenfeldi* to male rectal gland extract. Numbered peaks indicate active compounds: (*E*,*E*)-2,8-dimethyl-1,7-dioxaspiro[5.5]undecane (**3**) and (*E*,*E*)-2-ethyl-8-methyl-1,7-dioxaspiro[5.5]undecane (**4**)

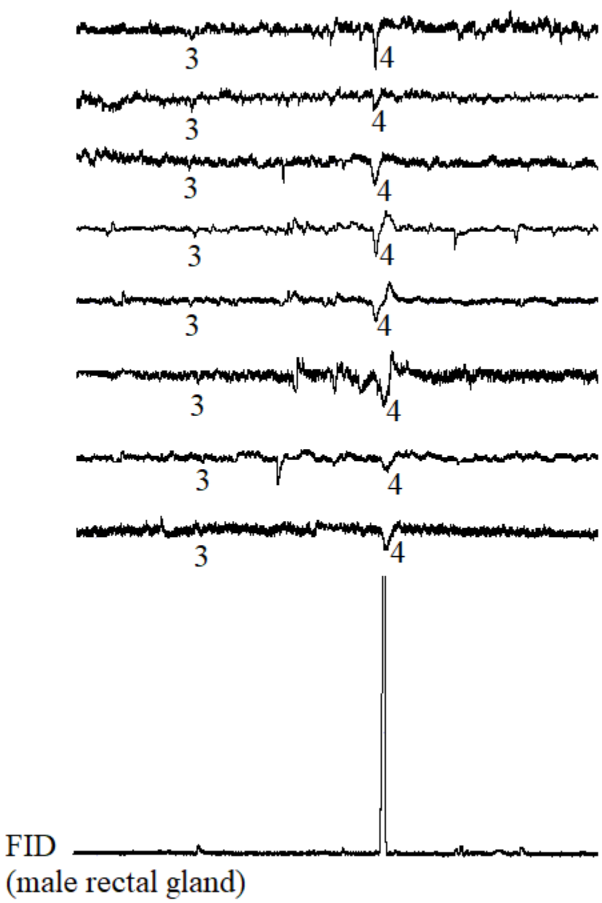

Figure S2. Female GC-EAD responses using male rectal gland.

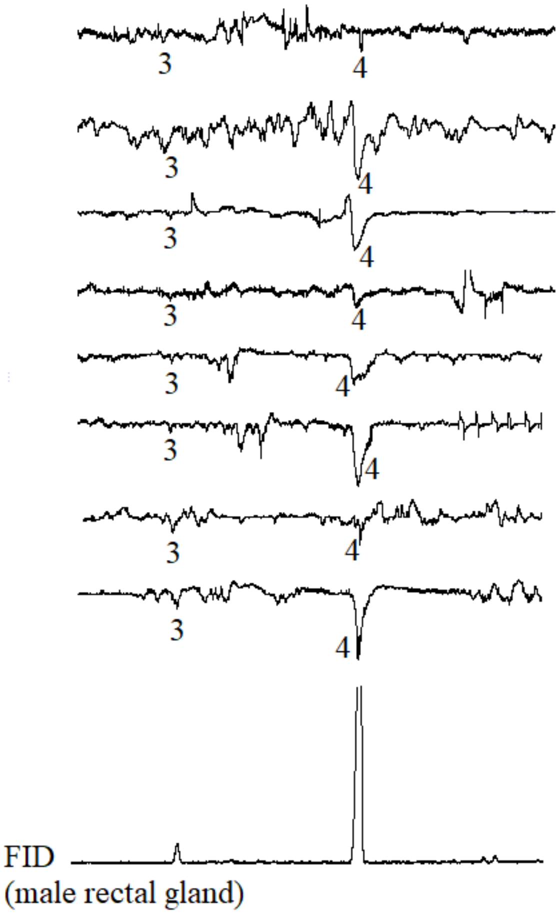

Figure S3. Female GC-EPD responses using male rectal gland.

Responses of male *B. frauenfeldi* antenna to male rectal gland extract. Numbered peaks indicate active compounds: (*E*,*E*)-2,8-dimethyl-1,7-dioxaspiro[5.5]undecane (**3**)

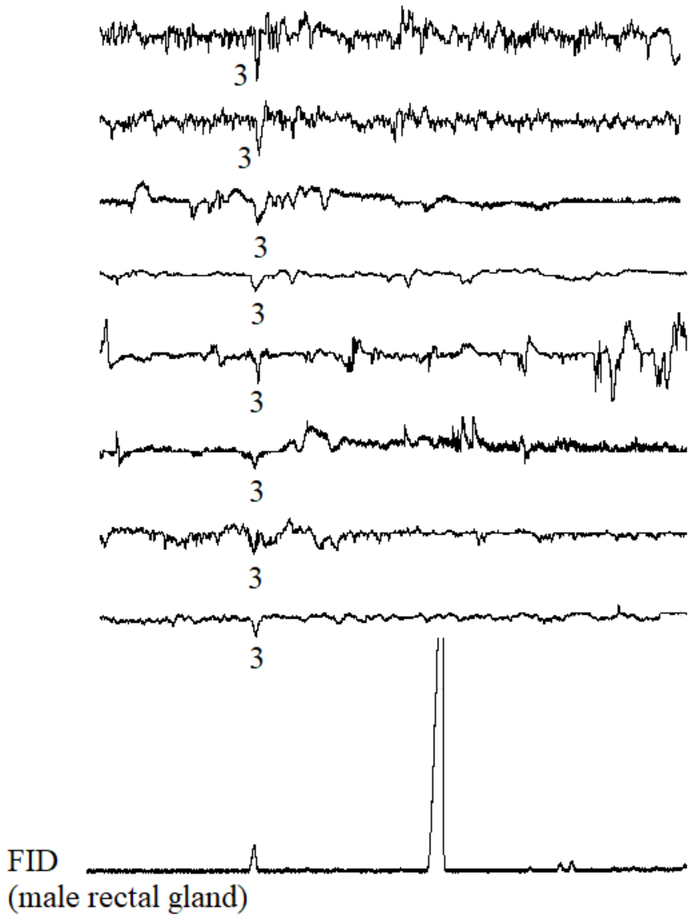

Figure S4. Male GC-EAD responses using male rectal gland.

Responses of male *B. frauenfeldi* maxillary palps to male rectal gland extract. Numbered peaks indicate active compounds: (*E*,*E*)-2,8-dimethyl-1,7-dioxaspiro[5.5]undecane (**3**) and (*E*,*E*)-2-ethyl-8-methyl-1,7-dioxaspiro[5.5]undecane (**4**)

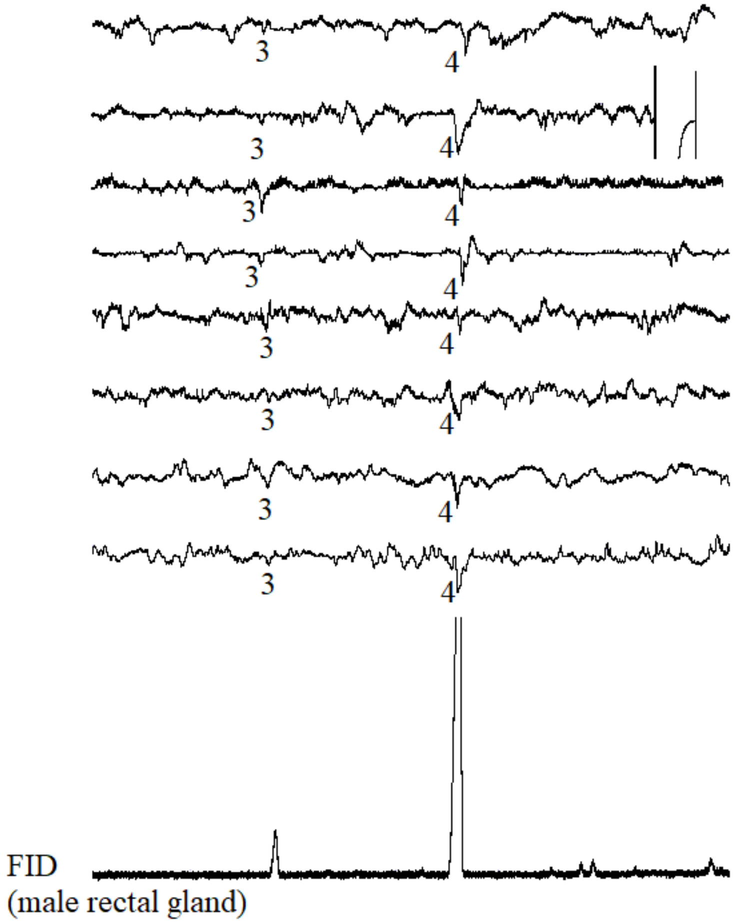

Figure S5. Male GC-EPD responses using male rectal gland.

Responses of female *B. frauenfeldi* antenna to female rectal gland extract. Numbered peaks indicate active compounds: (*E*,*E*)-2,8-dimethyl-1,7-dioxaspiro[5.5]undecane (**3**), (*E*,*E*)-2-ethyl-8-methyl-1,7-dioxaspiro[5.5]undecane (**4**) and ethyl caprate (**5**)

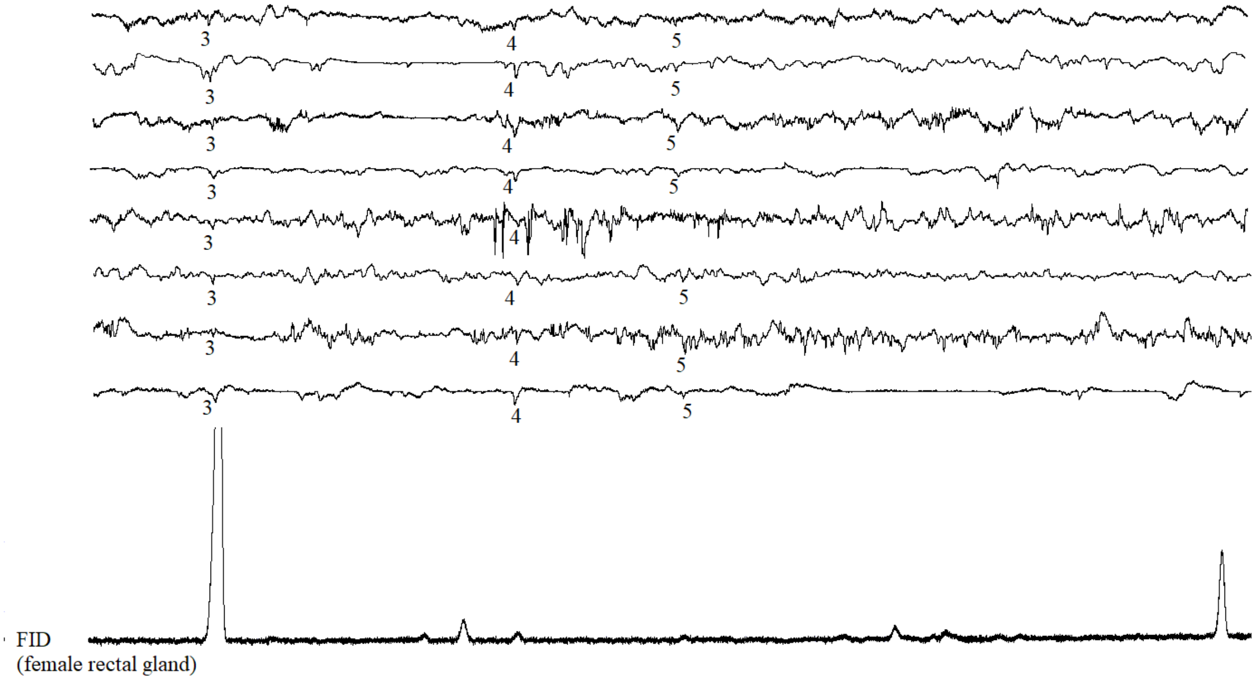

Figure S6. Female GC-EAD responses using female rectal gland.

Responses of female *B. frauenfeldi* palps to female rectal gland extract. Numbered peaks indicate active compounds: (*E*,*E*)-2,8-dimethyl-1,7-dioxaspiro[5.5]undecane (**3**), (*E*,*E*)-2-ethyl-8-methyl-1,7-dioxaspiro[5.5]undecane (**4**) and methyl laurate (**6**)

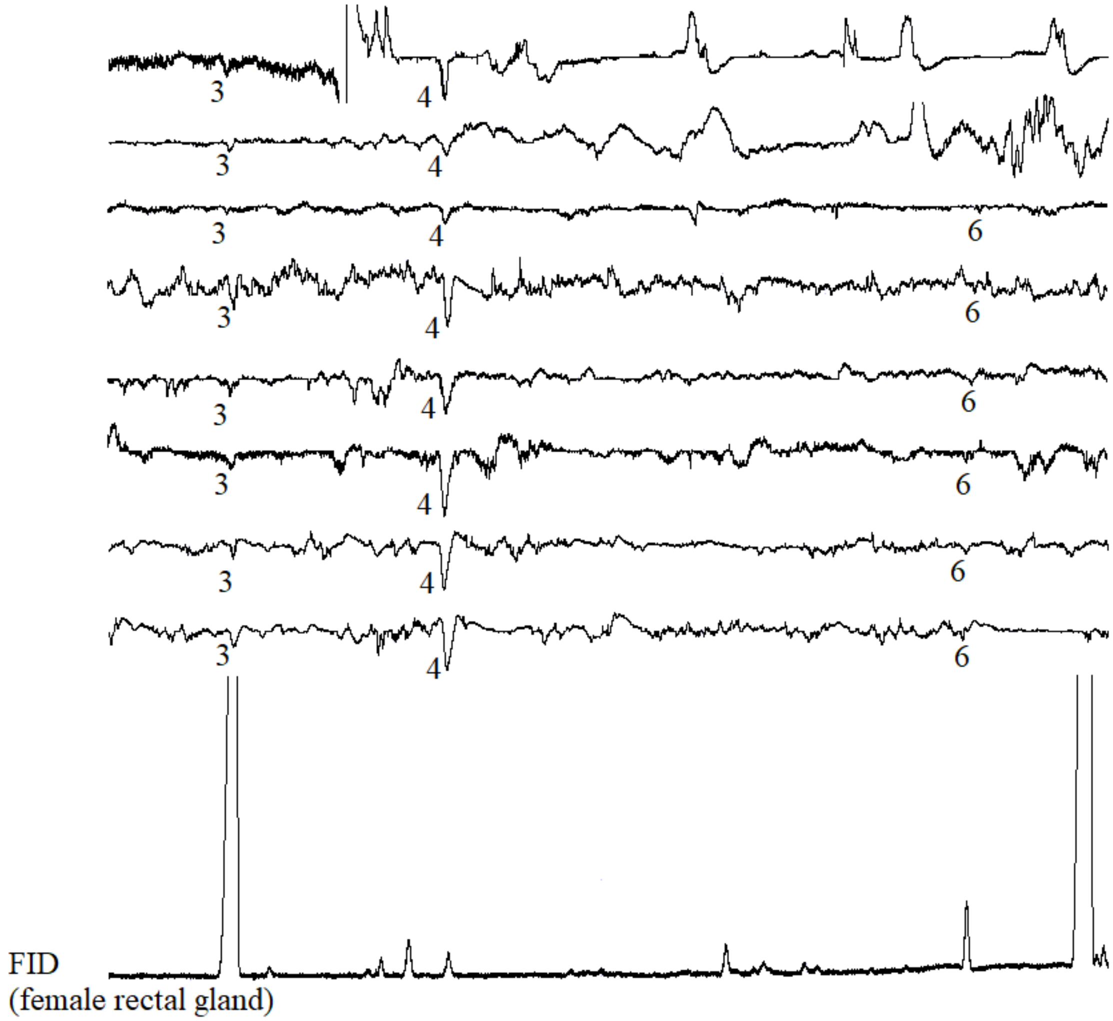

Figure S7. Female GC-EPD responses using female rectal gland.

Responses of male *B. frauenfeldi* antenna to female rectal gland extract. Numbered peaks indicate active compounds: (*E*,*E*)-2,8-dimethyl-1,7-dioxaspiro[5.5]undecane (**3**)

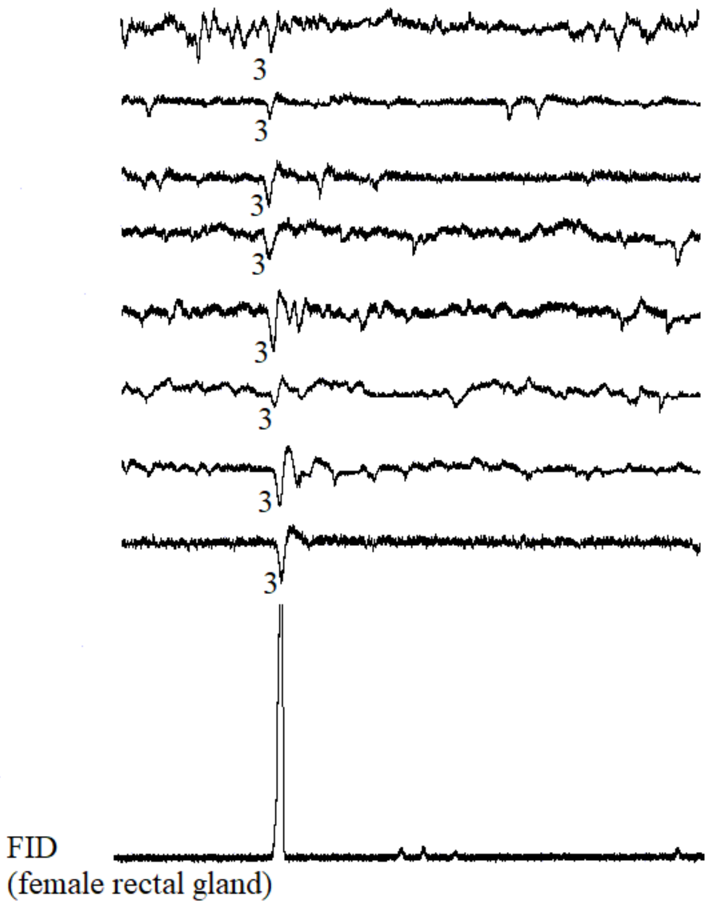

Figure S8. Male GC-EAD responses using female rectal gland.

Responses of male *B. frauenfeldi* maxillary palps to female rectal gland extract. Numbered peaks indicate active compounds: (*E*,*E*)-2,8-dimethyl-1,7-dioxaspiro[5.5]undecane (**3**) and (*E*,*E*)-2-ethyl-8-methyl-1,7-dioxaspiro[5.5]undecane (**4**)

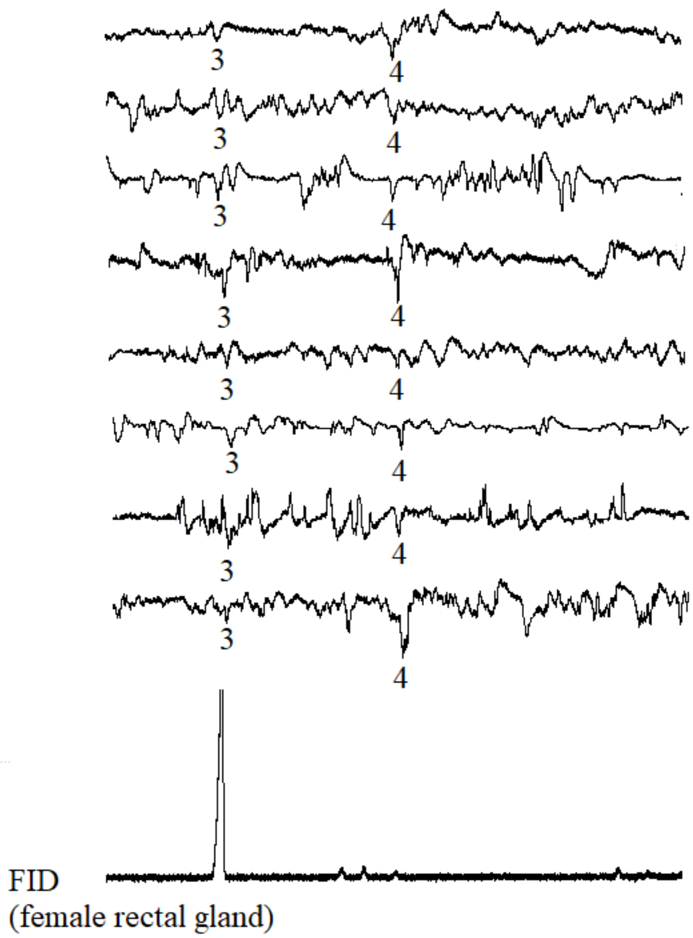

Figure S9. Male GC-EPD responses using female rectal gland.

References

1. Naik, S.; Bhattacharjya, G.; Talukdar, B.; Patel, B.K. Chemoselective acylation of amines in aqueous media. *European J. Org. Chem.* **2004**, *2004*, 1254–1260.
2. Wee, S.L.; Tan, K.H. Female sexual response to male rectal volatile constituents in the fruit fly, *Bactrocera carambolae* (Diptera: Tephritidae). *Appl. Entomol. Zool.* **2005**, *40*, 365–372.

3. Kitching, W.; Lewis, J.A.; Perkins, M. V; Drew, R.; Moore, C.J.; Schurig, V.; Koenig, W.A.; Francke, W. Chemistry of fruit flies. Composition of the rectal gland secretion of (male) *Dacus cucumis* (cucumber fly) and *Dacus halfordiae*. Characterization of (*Z*,*Z*)-2,8-dimethyl-1,7-dioxaspiro[5.5]undecane. *J. Org. Chem.* **1989**, *54*, 3893–3902.
4. Denton, R.M.; Tang, X.; Przeslak, A. Catalysis of phosphorus(V)-mediated transformations: dichlorination reactions of epoxides under Appel conditions. *Org. Lett.* **2010**, *12*, 4678–4681.
